# Risk Factors Associated with Preterm Neonatal Mortality: A Case Study Using Data from Mt. Hope Women’s Hospital in Trinidad and Tobago

**DOI:** 10.3390/children4120108

**Published:** 2017-12-14

**Authors:** Karen Cupen, Annabel Barran, Virendra Singh, Isaac Dialsingh

**Affiliations:** 1 Department of Mathematics and Statistics, The University of the West Indies, St. Augustine Campus, Trinidad and Tobago; karencupen@yahoo.com; 2 Department of Pediatrics, Faculty of Medical Sciences, The University of the West Indies, St. Augustine Campus, Trinidad and Tobago; belle_anna707@yahoo.com (A.B.); virendra.singh@sta.uwi.edu (V.S.)

**Keywords:** preterm neonatal mortality, neonatal intensive care unit, binary logistic regression, birth weight, length of time on the ventilator, Trinidad and Tobago

## Abstract

Preterm neonatal mortality contributes significantly to the high incidence of death among children under five years of age. Neonatal mortality also serves as an indicator of maternal health in society. The aim of the study is to examine the risk factors for preterm neonatal mortality at the neonatal intensive care unit (NICU) at Mount Hope Women’s Hospital in Trinidad and Tobago (MHWH). In this retrospective study, we included infants (*N* = 129), born < 37 weeks gestational age, between 1 January and 31 December 2015. Two binary logistic regression models (infant and maternal variables) were constructed to identify predictors of preterm neonatal mortality. Roughly 12% of the infants died after being admitted to the NICU. The binary logistic regression (infant model) had an excellent fit (area under the curve (AUC): 0.904, misclassification rate: 11.7%) whilst the maternal binary logistic model had a fair fit (AUC: 0.698). Birth weight, length of time on the ventilator and obstetric complications proved to significantly influence the odds of preterm neonatal death. The estimated models show that improvement in neonatal as well as maternal variables has direct impact on preterm neonatal mortality.

## 1. Introduction

Preterm birth is a significant factor in mortality among children who are under five years, as well as the leading cause of neonatal mortality due to the complications associated with preterm births [1]. Preterm birth has a pivotal role in the mortality and morbidity among newborns. In 2015, the World Bank reported that 85% of the neonatal mortality occurring around the globe is contributed by preterm birth [2]. This global trend is also seen in Trinidad and Tobago, as in 2008, the UNICEF outlined that among the under-five year olds, 69% of the deaths was attributed to neonatal mortality, of which prematurity was responsible for 31% of the neonatal deaths [3].

Blencowe et al. reported that not only is preterm birth a major cause of mortality, but also a major contributor to loss of human potential due to neonatal morbidities [4]. There is significant morbidity among some survivors including weakening neurodevelopment capacities, learning impedance, visual disorders and secondary effects in long term health [5,6,7]. These morbidities have substantial consequences to the families, society and the health system [8].

Preterm birth can be classified based on gestational age as extremely preterm (less than 28 weeks), very preterm (between 28 and 32 weeks), moderate (between 32 and 34 weeks), and late preterm (34 to less than 37 weeks) [9]. Preterm birth is categorized as either spontaneous by preterm premature rupture of membranes (pPROM) or provider-initiated, which includes elective caesarian preterm birth [10].

There have been a number of previous studies attempting to identify the risk factors associated with preterm birth in different countries. In a study conducted by Stoll et al. the rate of survival to discharge was directly proportional to gestational age [10]. Similar studies showed that small for gestational age [11] and low birth weight [12] are associated with increased risk of neonatal mortality.

Bin Pak and Horiuchi found no significant relationships between maternal factors (i.e., age and race) and preterm birth [12]. However, Mengesha et al. noted that underweight maternal body mass index and gravidity were related to preterm births [13]. In other studies conducted in Denmark, Nigeria, Switzerland, and South Africa risk factors such as alcohol consumption, low socioeconomic status, suburban areas, resource-limited health care (maternal and neonatal) were associated with preterm birth [14,15,16,17].

A comparison analysis of child and infant mortality, including Latin America and the Caribbean, revealed that Trinidad and Tobago is falling behind its regional counterparts, even though these countries have smaller per capita incomes and lower per capita health [5]. Table 1 illustrates the neonatal mortality rate of countries in the Caribbean. This rate is calculated as the number of newborns who die before 28 days in every 1000 live births in a particular year [4]. Trinidad and Tobago has a much higher neonatal mortality rate compared to most Caribbean countries.

In Trinidad and Tobago, routine data on preterm births are usually not collected and if so, are rarely reported. This analysis will help in understanding the factors that are related to preterm neonatal mortality. Considering the high preterm rate as well as the relatively higher mortality, there is an urgent need to conduct a research that would guide medical practitioners, care-givers, policy-makers and the Government in drawing effective plans and in making informed decisions with regard to preterm neonatal morbidity and mortality.

## 2. Materials and Methods

This study examined the neonates delivered at gestational age less than 37 weeks and admitted to Mount Hope Women’s Hospital in Trinidad and Tobago (MHWH) neonatal intensive care unit (NICU) for the period 1 January–31 December 2015. All medical variables were identified by their standard accepted and practiced usage. It must be noted that the data collection was manual in nature and not all the variables from other studies in the literature were available. In addition, sometimes the numbers were quite small. There were 151 preterm births, however, 129 complete birth records were available for the study. Perinatal and neonatal data were collected retrospectively from available patient records stored at MHWH using preformed data collection sheets by pediatric trained doctors.

The neonatal variables incorporated gender (GEN), gestational age (GES), birth weight (BWG), necrotizing enterocolitis (NEC) (as evidenced by the presence of air within the bowel wall), and the length of time on the ventilator (LTV), whilst the maternal variables were maternal age (MAT), gravidity (GRA) and obstetric complications (COM).

The data were entered, coded and analyzed using SPSS (version 20.0, IBM Corp., Armonk, NY, USA) computer software program. Binary logistic regression was done. All variables listed above were binary with the exception of length of time on the ventilator. *p*-value < 0.05 was taken as statistically significant. Confidence intervals were also calculated.

The Receiver Operating Characteristic (ROC) curve is a useful way to assess the performance of classification schemes in which there is one variable which is classified into two categories [1]. The area under the ROC curve measures the discrimination, i.e., the ability to categorize those who survive and those who do not. An area of 1 represents a perfect test and an area of 0.5 represents a worthless test [18].

Ethical approval and clearance were obtained from the University of the West Indies as well as the North Central Regional Health Authority since this analysis used entirely registered data (CEC047/11/15). Furthermore, confidentiality and anonymity were assured by analyzing and disseminating the findings in aggregate.

## 3. Results

Preterm births (<37 weeks gestation) in this population consisted of 59% males and 41% females. Of these, 76 (59.7%) were moderate to late preterm (33 to <37 weeks), 43 (33.3%) were very preterm (28 to <32 weeks) and 9 (7.5%) were extremely preterm (<28 weeks). Birth weight ranged from 725 to 2900 g (standard deviation (SD): 485.6 g), maternal age from 17 to 43 years (SD: 6.6 years) and gravidity from 1 to 8 pregnancies (SD: 1.7 pregnancies).

Among the neonates, the length of time on the ventilator ranged from 1 to 71 days. However, the length of stay ranged from 1 to 164 days (SD: 24.4 days) with an average of 21.7 days. Overall, 16 infants (12.4%) died before being discharged, and 11.6% (15) survived with disability evident at discharge.

### 3.1. Cause of Death among Children

Table 2 summarizes the major causes of death among the 16 children who died prematurely. Some of these children had multiple cause of deaths on their death certificate. Among the 16 children who died prematurely, ELBW (Extremely Low Birth Rate) and prematurity each accounted for 75% (12 out of 16) of the cause of death. Sepsis/infections as well as Pneumothorax each accounted for 8.3% of deaths and 31.3% had sepsis/infections and pneumonia as a cause of death on their death certificate. Pulmonary hemorrhage accounted for 6.7% of deaths with 31.3% of patients having this listed as a cause of death on their death certificate.

Among the maternal and neonatal variables considered, only neonatal variables were significantly correlated with preterm neonatal mortality i.e., birth weight, gestational age and respiratory distress syndrome. The results for the univariate analysis of maternal and neonatal characteristics of the subjects in relation with preterm neonatal mortality are summarized in Table 3.

The list of obstetric complications was also analyzed and is shown in Table 4.

Binary logistic regression was done to show the association between maternal and neonatal variables in relation to preterm neonatal mortality.

### 3.2. Binary Logistic Regression for Neonatal Variables

Based on the results obtained using neonatal variables: length of time on the ventilator (Odds Ratio (OR) = 1.09, Confidence Interval (CI): 1.03–1.15, *p* = 0.004) and very low to extremely low birth weight (OR = 15.41, CI: 2.00–120.34, *p* = 0.01) were identified as significant risk factors for preterm neonatal mortality (Table 5).

**Figure 1:**
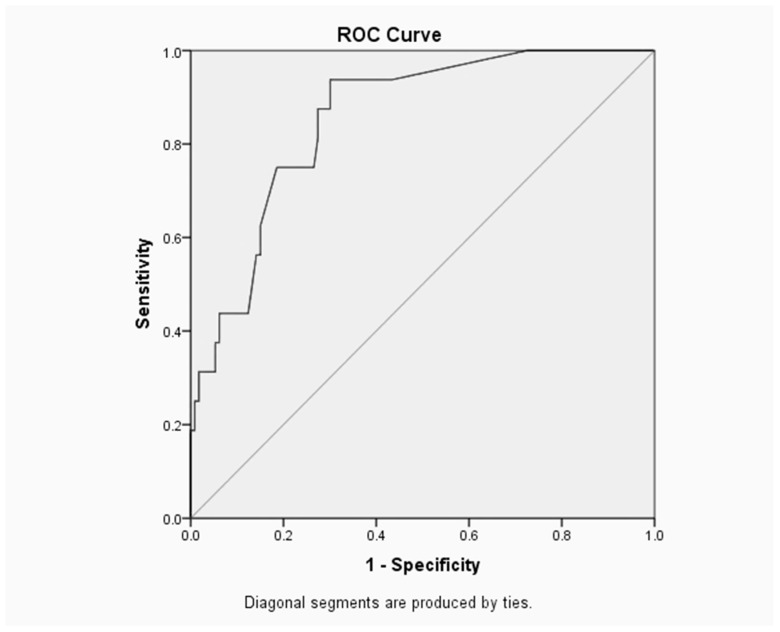
Receiver Operating Characteristic (ROC) Curve and Area for the Infant Binary Logistic Regression Model.

The area under the ROC curve is 0.904 as shown in Figure 1.

#### Neonatal Model

The binary logistic regression model for the neonatal variables is

(1) Logit (neonatal mortality)=−4.31−0.38GEN+0.25BWG+0.11NEC+0.09LTV

where *GEN* = 0.1; *GES* = 0.1; *BWG* = 0.1; *NEC* = 0.1.

### 3.3. Binary Logistic Regression for Maternal Variables

Table 6 illustrates the multivariate binary logistic regression for the maternal variables. Obstetric complications (≥1) (OR = 8.73, CI: 1.07–71.09, *p* = 0.04) was the only significant risk factor for preterm neonatal mortality.

**Figure 2:**
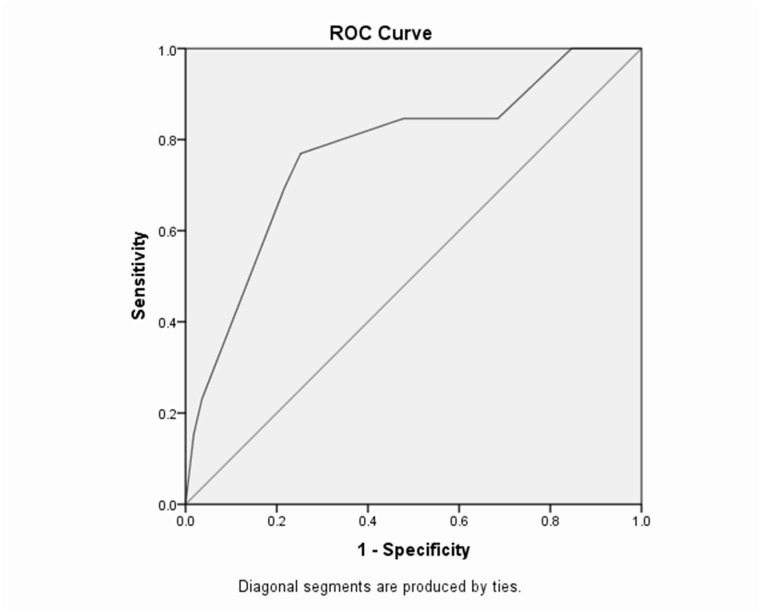
ROC Curve and Area for the Maternal Binary Logistic Regression Model.

The area under the ROC curve is 0.698 as shown in Figure 2.

#### Maternal Model

The binary logistic regression model for the maternal variables is

(2) $$Logit\;\left(neonatal\;mortality\right)=-3.72+0.266MAT_{19–35}-0.15MAT_{>35}+2.17COM-0.2GRA$$

where *MAT* = 0.1; *COM* = 0.1; *GRA* = 0.1.

## 4. Discussion

The investigation utilized the binary logistic regression and the study encompassed all neonates delivered at gestational age less than 37 weeks and admitted to the NICU at MHWH for the year 2015. There were 151 preterm births, however, 129 complete birth records were available for the study.

The area under the ROC curve (Figure 1) is 0.904, which suggests that the neonatal model provided an excellent fit. The optimal sensitivity/specificity point is located in the uppermost left corner on ROC curve, thus the optimal sensitivity is 93.8% and specificity (1–0.221) is 77.9%.

However, for the maternal model, the area under the ROC curve (Figure 2) is 0.698 which represents a fair test. Furthermore, the optimal sensitivity/specificity point is 90.9% for sensitivity and 46.4% (1–0.536) for specificity.

The results achieved from this single tertiary centre study will provide information about preterm neonatal mortality and will aid in achieving the millennium development goal of reducing under-five mortality by 2015 and beyond [19]. Even though similar studies may have been conducted elsewhere, the difference in hospital policies, availability of resources and clinical practices among hospitals and countries will make the conclusions of those studies irrelevant to the Trinidad and Tobago experience.

Finally, the research will hopefully provide relevant information that may serve as a starting basis for those who may wish to conduct further as well as longer term research, relating to preterm neonatal morbidity and mortality.

Limitations of the study entailed its small sample size and the short time period of one year for the study. Nonetheless, this is the first step in the interpretation and statistical analysis of the data with relevance to the country of Trinidad and Tobago. The logistic regression model for the neonatal variables comprised of gender, gestational age, birth weight, necrotizing enterocolitis and the length of time on the ventilator. This study demonstrated that the log-odd value, β, for birth weights < 1500 g (*p* = 0.010) relative to birth weights between 1500 g and 2500 g is 2.74, i.e., the neonatal mortality is inversely proportional to birth weight. The latter result is similar to previous studies [9,11,17].

In 2007, the Institute of Medicine (U.S.) Committee reported that infants born before 30 weeks gestation lacked alveoli, as a result of underdeveloped lungs there was the need for assistance to breathe [19]. The present study found that the length of time on the ventilator (*p* = 0.004) increased the predicted counts of mortality by 1.09. Poor postnatal lung function resulted in both increase morbidity, and that increased length of time on the ventilator also having an adverse association with neonatal outcome.

According to a similarly represented study by Onuanaku et al., gender is not a significant predictor of neonatal mortality [20], this result was also found in the MHWH data set. However, the odd ratio for male neonates was 0.68, this indicates that male (preterm) neonates have a lower odds of mortality relative to female (preterm) neonates.

Although the leading cause of death in preterm births is NEC [21], our study did not reproduce this result (NEC (*p* > 0.05)). This result may be driven by the considerably low neonatal deaths (*n* = 16) in our data set. However, the logit model predicted an 11% increase in mortality for neonates born with NEC relative to those without the disease.

The number of obstetric complications proved to be the only significant predictor of mortality in the maternal model (maternal age, gravidity and obstetric complications). Yego et al. found that the risk of early neonatal death was higher for mothers who experienced PROM, haemorrhage and dystocia whereas the other cases of complications (HIV, malaria, previous scar, retained placenta and anaemia) had no significant effect [22]. Our study differed from Yego et al.’s. The complications that were most commonly found and subsequently explored were pPROM, increased body mass index, diabetes mellitus, pre-eclampsia, eclampsia, hypertension and smoking during pregnancy. Nonetheless, similar results were yielded, i.e., mothers with one or more complications relative to mothers without complications, the log-odd value, β, for neonatal mortality increased by 2.17.

According to Bin Pak and Horiuchi maternal age is not a neonatal mortality risk factor; our study also observed the same result [12]. The study by Aras concluded that mothers (aged 40 and up) were at high risk for preterm births [23]. In contrast, our study reported that mothers (aged 35 and up) had a lower odd ratio (OR = 0.86, 95% CI: 0.16, 4.62, *p* = 0.86) relative to mothers (age < 19) in association with neonatal mortality. Births to mothers between 19 and 35 years had a higher risk (OR = 1.30, 95% CI: 0.08–21.36, *p* = 0.85) of mortality compared to the reference category in this data set. This is indeed unusual and this may be an artifact of our small sample size. This study is preliminary in nature and provides the foundation for further work to address these perceived anomalies.

According to Kurdi et al. preterm birth remains the most serious complication of multiple gestations [24]. In our study, however, gravidity was statistically insignificant (*p* = 0.77) as a determinant of preterm neonatal death, the latter results were also reported by Yego et al.

The strength of our analysis is the use of the outcome information collected before or around the time of birth. There are some limitations of the study: missing neonates’ records, the time period of one year and the consequent small sample size.

A valuable extension to this study is to include other NICUs in Trinidad and Tobago. Table 2 summarizes the main cause of death among newborns of perinatal risk factors for neonatal/morbidity from the 16 children who died prematurely. This list will serve as a basis for the variables that need to be collected prospectively in the future. This would give us a more comprehensive picture of how to proceed with future studies. 

A longitudinal study will also be able to reveal trends. Furthermore, it will be interesting and useful for future studies to include individual obstetric complications especially those related to lifestyles. Finally, this study presents a snapshot of the present state of affairs at one of the hospitals in Trinidad and Tobago. We are cognizant of the fact that the results of the study cannot be generalized to the entire population of newborns in Trinidad and Tobago. However, the study does present sufficient information to make decision makers more aware of the factors that contribute to mortality in the NICU at MHWH. This can lend itself to better resource allocation and preventative measures being instituted.

## 5. Conclusions

The neonatal variables birth weight and length time on the ventilator, as well as obstetric complications (maternal variable), proved to be risk factors of preterm neonatal mortality. However, gender, gestational age, necrotizing enterocolitis, maternal age and gravidity proved to be insignificant variables. Further work needs to be carried out to address these unusual occurrences.

The neonatal and maternal models can be useful in providing useful descriptive information with regard to outcome expectations of the neonates admitted to the NICU at MHWH.

## Figures and Tables

**Table 1:** List of Caribbean Countries by Neonatal Mortality Rate per 1000 live births.

Country	Rate
Antigua and Barbuda	5
Barbados	8
Cuba	2
Dominica	16
Grenada	6
Jamaica	12
St. Kitts and Nevis	7
St. Lucia	9.3
St. Vincent and the Grenadines	12
Trinidad and Tobago	13

**Table 2:** Major causes of death in children who died.

		Responses		Percent of Cases
		*N*	Percent	
Cause of Death	Prematurity	12	20.0%	75.0%
	ELBW	12	20.0%	75.0%
	Pneumonia	10	16.7%	62.5%
	Pulmonary Haemorrhage	4	6.7%	25.0%
	Sepsis/infections	5	8.3%	31.3%
	Pneumothorax	5	8.3%	31.3%
	NEC	4	6.7%	25.0%
	Birth Asphyxia	1	1.7%	6.3%
	Hypotension	1	1.7%	6.3%
	CLD	1	1.7%	6.3%
	Multi-Organ Failure	1	1.7%	6.3%
	IVH	2	3.3%	12.5%
	Thrombocytopenia	1	1.7%	6.3%
	Other	1	1.7%	6.3%
Total		60	100.0%	

ELBW: extremely low birth weight; NEC: necrotizing enterocolitis; CLD: chronic lung disease; IVH: intraventricular haemorrhage.

**Table 3:** Univariate analysis of base characteristics to assess predictors of preterm neonatal mortality.

Variables	Survived		Total 129	OR & 95% CI for OR	*p*-Value
	Yes (%)	No (%)			
Gender					
Female	42 (42.4)	5 (31.3)	53	1.62 (0.53, 4.98)	0.39
Male	65 (57.6)	11 (68.7)	76	1	
Birth Weight, grams					<0.0001 ***
<1000	2 (1.8)	9 (56.2)	11	0.01 (0, 0.04)	
1000–1500	30 (27.0)	5 (31.3)	35	0.15 (0.03, 0.83)	
>1500	75 (66.3)	2 (12.5)	81	1	
Gestational Age, weeks					<0.0001 ***
<28	3 (2.7)	6 (37.5)	9	0.01 (0, 0.1)	
28–32	35 (31.0)	8 (50.0)	43	0.12 (0.02, 0.58)	
33–37	75 (66.3)	2 (12.5)	77	1	
Maternal Age, years					0.87
<19	8 (7.2)	1 (9)	9	0.76 (0.06, 9.61)	
19–35	82 (73.9)	8 (73.0)	90	0.98 (0.19, 4.94)	
>35	21 (18.9)	2 (8.0)	23	1	
NEC					
Yes	15 (13.2)	3 (18.8)	18	0.66 (0.17, 2.59)	0.69
No	98 (86.8)	13 (81.2)	111	1	
RDS					
Yes	32 (28.3)	13 (81.2)	45	0.09 (0.02, 0.33)	<0.0001 ***
No	81 (71.7)	3 (18.8)	84	1	
Gravidity					
=1	42 (37.8)	6 (46.2)	48	0.71 (0.22, 2.27)	0.56
>1	69 (62.2)	7 (53.8)	76	1	

OR: odds ratio; CI: confidence interval; RDS: respiratory distress syndrome; *** *p* ≤ 0.001.

**Table 4:** Univariate analysis of obstetric complications to assess predictors of preterm neonatal mortality.

Variables	Survived		Total 129	OR & 95% CI for OR	*p*-Value
	Yes (%)	No (%)			
Cervical competence					
No	111 (88.1)	15 (11.9)	126	1	
Yes	2 (66.7)	1 (33.3)	3	3.7 (0.32, 43.32)	0.27
Hypertension					
No	99 (89.2)	12 (10.8)	111	1	
Yes	14 (77.8)	4 (22.2)	16	2.3 (0.67, 8.33)	0.17
Pre-eclampsia					
No	103 (88.8)	13 (11.2)	116	1	
Yes	10 (76.9)	3 (23.1)	13	2.4 (0.58, 9.77)	0.22
Diabetes Mellitus					
No	99 (86.1)	16 (13.9)	115	1	
Yes	14 (100)	0 (0)	14	0.8 (0.80, 0.98)	0.14
Eclampsia					
No	105 (87.5)	15 (12.5)	120	1	
Yes	8 (88.9)	1 (11.1)	9	0.9 (0.10, 1.49)	0.90
Increased BMI					
No	111 (88.1)	14 (11.2)	125	1	
Yes	2 (66.7)	1 (33.3)	3	3.7 (0.32, 43.32)	0.27
Reverse End Diastolic Flow					
No	111 (88.8)	14 (11.2)	125	1	
Yes	2 (50)	2 (50)	4	7.3 (1.03, 60.8)	0.02 **
pPROM					
No	84 (87.5)	12 (12.5)	96	1	
Yes	29 (87.9)	4 (12.1)	33	1.0 (0.31, 3.47)	1.00

** *p* ≤ 0.05; BMI: body mass index; pPROM: preterm premature rupture of membranes.

**Table 5:** Multivariate binary logistic regression output for neonatal variables.

Variables	β	SE β	Wald’s	OR & 95% for OR	*p*-Value
Constant	−4.31	1.20	12.86		<0.001 **
Gender					
Female				1	-
Male	−0.38	0.71	0.29	0.68 (1.71, 2.78)	0.74
Gestational Age					
Moderate to Late Preterm				1	-
Extremely to Very Preterm	0.25	0.75	0.11	1.29 (0.30, 5.54)	0.74
Birth Weight					
Low Birth Weight				1	-
Very Low to Extremely Low Birth Weight	2.74	1.05	6.80	15.41 (2.00, 120.34)	0.01 **
Necrotizing Enterocolitis					
No				1	-
Yes	0.11	0.79	0.02	1.11 (0.27, 5.17)	0.90
Length of Time on the Ventilator	0.09	0.03	8.28	1.09 (1.03, 1.15)	0.004 **

β: regression coefficient in the multivariate analysis; SE β: standard error; ** *p* ≤ 0.05.

**Table 6:** Multivariate binary logistic regression output for maternal variables.

Variables	β	SE β	Wald’s	OR & 95% for OR	*p*-Value
Constant	−3.73	1.35	7.67	-	0.01 **
Maternal Age, years			1.38	-	0.93
<19				1	-
19–35	0.27	1.43	0.04	1.30 (0.08, 21.36)	0.85
>35	−0.15	0.86	0.03	0.86 (0.16, 4.62)	0.86
Gravidity					
=1				1	-
>1	−0.20	0.70	0.90	0.82 (0.21, 3.16)	0.77
Obstetric Complication					
=0				1	-
≥1	2.17	1.07	4.10	8.73 (1.07, 71.09)	0.04 **

** *p* ≤ 0.05.
